# Living With Inflammatory Bowel Disease: Protocol for a Longitudinal Study of Factors Associated With Symptom Exacerbations

**DOI:** 10.2196/11317

**Published:** 2018-11-12

**Authors:** Kelcie Witges, Laura E Targownik, Clove Haviva, John R Walker, Lesley A Graff, Kathryn A Sexton, Lisa Lix, Michael Sargent, Kathy Vagianos, Charles N Bernstein

**Affiliations:** 1 IBD Clinical and Research Centre University of Manitoba Winnipeg, MB Canada; 2 Department of Medicine University of Manitoba Winnipeg, MB Canada; 3 Department of Psychology University of Manitoba Winnipeg, MB Canada; 4 Department of Clinical Health Psychology University of Manitoba Winnipeg, MB Canada; 5 Department of Community Health Sciences University of Manitoba Winnipeg, MB Canada

**Keywords:** Crohn disease, diet, flare, inflammatory bowel disease, survey, ulcerative colitis, Web-based

## Abstract

**Background:**

There has been limited longitudinal research that has comprehensively evaluated possible factors in the exacerbation of inflammatory bowel disease (IBD) symptoms with or without associated inflammation. Evolving Web-based technologies facilitate frequent monitoring of patients’ experiences and allow a fine-grained assessment of disease course.

**Objective:**

We aimed to prospectively identify factors associated with symptom exacerbation and inflammation in IBD including psychological functioning, diet, health behaviors, and medication adherence.

**Methods:**

Between June 2015 and May 2017, we enrolled adults with IBD, recruited from multiple sources, who had been symptomatically active at least once within the prior 2 years. They completed a Web-based survey every 2 weeks for 1 year and submitted a stool sample at baseline, 26 weeks, and 52 weeks. Any participant reporting a symptom exacerbation was matched to a control within the cohort, based on disease type, sex, age, and time of enrollment; both were sent a supplemental survey and stool collection kit. Biweekly surveys included validated measures of the disease course, psychological functioning, health comorbidities, and medication use. Intestinal inflammation was identified through fecal calprotectin (positive level >250 μg/g stool).

**Results:**

There were 155 participants enrolled with confirmed IBD, 66.5% (103/155) with Crohn disease and 33.5% (52/155) with ulcerative colitis, of whom 98.7% (153/155) completed the study. Over the 1-year period, 47.7% (74/155) participants experienced a symptom exacerbation. The results of analyses on risk factors for symptom exacerbations are pending.

**Conclusions:**

We recruited and retained a longitudinal IBD cohort that will allow the determination of risk factors for symptom exacerbation with and without inflammation. This will increase understanding of symptom exacerbations among persons with IBD.

**International Registered Report Identifier (IRRID):**

RR1-10.2196/11317

## Introduction

Inflammatory bowel disease (IBD), manifesting as Crohn disease (CD) or ulcerative colitis (UC), is complex. Individuals experience gastrointestinal symptoms such as abdominal pain, diarrhea, and rectal bleeding, but they may also have fatigue or extraintestinal manifestations such as arthritis [1]. As with many chronic illnesses, the clinical impact of IBD cannot be fully accounted for with objective tests, such as endoscopy or biological markers [2]. Psychological factors and diet have been identified as contributing to symptoms and patient outcomes [3-6]. To better understand the role of biological, psychological, and lifestyle factors in the expression and course of IBD, there is a need to prospectively study these multiple domains.

People living with IBD often face episodes of increased symptom activity separated by periods of relative or absolute quiescence. We have shown that 30% of individuals with IBD who report no symptoms will become symptomatic at some time over the following year [4]. People often report a lower health-related quality of life [7] during these periods, and many may seek medical care for the management of these symptoms [8]. Importantly, not all symptoms in patients with IBD are related to active inflammation, although most IBD therapies are directed at reducing inflammation [4,9].

There has been little research exploring the causes of the development or worsening of IBD-related symptoms and what exactly it means when a patient reports a “flare.” Persons with IBD often attribute symptom exacerbation to changes in diet and stress, although there is currently little understanding of which dietary or psychological factors play a role. Symptomatic activity in IBD is generally reported in clinical studies using tools such as the Harvey-Bradshaw Index that have poor biopsychometric properties [10]. Further, current Web-based technologies allow fine-grained assessment of individuals’ disease experience in a cost-effective and efficient manner [11].

This study aims (1) to assess patients’ experiences of symptomatic activity every 2 weeks over a 1-year period; (2) to describe which specific symptoms are correlated with patient-defined IBD flares, which are correlated with inflammation measured by fecal calprotectin (FCAL), and what patients believe causes their symptom exacerbations; (3) to identify predictors of patient-reported symptom exacerbations and, separately, inflammation, including psychological characteristics (perceived stress, self-efficacy, distress, optimism, intolerance of uncertainty, and health anxiety), diet, menstrual cycle, medication, comorbidity, and substance use; and (4) to describe diet in IBD, including stability over 1 year, and whether diet changes before or after symptom exacerbation.

In this report, we describe the protocol, participant enrollment, and participant retention for the study. Having recently completed the data collection, we are ready to undertake our varied analyses, which will be the subject of future reports.

## Methods

### Recruitment

Participants with established IBD were recruited from a previous longitudinal cohort study [12], our provincial population-based research registry [13], regional gastroenterology clinics, posters in the hospitals and gastroenterologists’ offices, and information posted on our center’s website (ibdmanitoba.org). Diagnoses of IBD were confirmed by medical records review and by querying treating physicians directly as needed. All recruitment was undertaken using a standardized script either in person or via email.

Inclusion criteria were (1) being aged 18-75 years old; (2) being able to provide informed consent; (3) being able to complete Web-based surveys in English; (4) willing to participate in the study for 1 year; and (5) having experienced symptoms related to IBD within the past 2 years.

Exclusion criteria were (1) total colectomy, ileostomy, or colostomy; (2) inadequately characterized disease history (disease phenotype and duration); (3) taking immune-modulating medication for any condition not related to IBD; (4) cancer treatment in the last 2 years (breast cancer patients on long-term tamoxifen who were well were included); and (5) specific serious chronic diseases other than IBD (ie, insulin-dependent diabetes, cirrhosis unless currently well, dialysis-dependent renal failure, organ transplantation, HIV, dementia, lupus, and scleroderma).

### Participant Retention

Reminder emails were sent at 24, 48, and 72 hours if the biweekly survey had not been completed. Telephone contact was made with participants at 72 hours if the survey was not completed. Participants received an honorarium of Can $10 for each completed survey and an additional Can $50 for successfully completing at least 80% of all surveys and 100% of requested stool specimens, to a maximum honorarium of Can $320.

### Data Collection

Long surveys were administered at baseline, midpoint (week 26), and study completion (week 52), while short surveys were completed every 2 weeks. At weeks 2 and 50, the short surveys were supplemented with a food frequency questionnaire. Quarterly, supplemental surveys added detailed medication and IBD clinical investigation questions. Stool specimens were collected for FCAL measurement at baseline, midpoint, and study completion.

Participants were sent an additional survey when they reported at least moderate worsening in symptom activity, as assessed by the IBD Symptom Change Indicator (SCI). Each of these cases was then matched with a control who had not previously experienced a symptom exacerbation and was not currently experiencing an exacerbation. Controls were matched on age (within 5 years), disease type, sex, and study entry date (to control for the season). Controls answered a similar additional survey. Cases and controls were also asked to return a stool collection kit. A maximum of 5 stool samples was collected from each participant over the year of the study (weeks 0, 26, and 52; up to 1 control; and up to 2 symptom exacerbations). Survey data were collected and managed using REDCap electronic data capture tools, hosted at the University of Manitoba [14].

### Measures

The measures against which data were collected include background characteristics, health behaviors, psychological qualities, IBD symptom activity and health-related outcomes, medication, and possible triggers of symptom exacerbation. If not otherwise stated, these were measured every 2 weeks. See Multimedia Appendix 1 for a tabular representation of when each measure was administered.

### Background Characteristics

#### Demographics

At baseline, participants reported their (1) sex; (2) date of birth; (3) marital status: single or never married, married or living as married, separated, divorced, widow(er); (4) employment status: working full time, working part-time, homemaking, attending school, both working and attending school, retired, disabled, or other; (5) ethnic background: Canadian, indigenous or Aboriginal, European, Jewish, Latina or Latino or Hispanic, Asian, African or black, other, or do not know or prefer not to answer (participants could choose all ethnicities that were applicable); (6) education: grades 1-13, apprenticeship, nonuniversity program (college, technical, business, vocational, and nursing), university program; and (7) annual household income (in Canadian dollars): <$25,000, $25,001-$50,000, $50,001-$75,000, $75,001-$100,000, $100,001-$150,000, >$150,000, or do not know or prefer not to say.

#### Inflammatory Bowel Disease Characteristics

At baseline, participants reported (1) IBD disease type as CD, UC, unclassified or indeterminate colitis, or do not know or not sure; (2) the year they were diagnosed with IBD; (3) their IBD-related surgeries (number, type, and year); and (4) whether they had been hospitalized for IBD over the previous 12 months.

#### Medical History

Participants reported at baseline whether a doctor had ever diagnosed them with any chronic condition and whether they had undergone any non-IBD surgery. Biweekly, they reported if any chronic illness (other than IBD) had worsened in the last 2 weeks or if a physician had diagnosed or treated them for any new condition, including minor illnesses or injuries (eg, sprained ankle). Menstruating women (according to the baseline report) were asked if their period had started in the last 2 weeks. At the end of the study, female participants who had not experienced menopause reported if they had become pregnant in the last year. If they had, they were asked to specify the start and end dates of the pregnancy.

#### Smoking Cigarettes and Drinking Alcohol

At baseline, participants reported smoking status as daily or almost daily, occasionally, former, or never. Alcohol consumption was reported as the average number of drinks per week.

#### Diet

Diet was assessed using the 2007 Harvard Food Frequency Questionnaire [15]. Food frequency questionnaires (FFQs) are a validated dietary assessment tool for epidemiological research [15]. The FFQ is a checklist of foods with a section for reporting how often each food item was consumed. The Harvard FFQ, with results generally stable over a year, was administered at weeks 2 and 50. Food categories included dairy, fruits, vegetables, eggs and meats, sweets and baked goods, dietary supplements, beverages, and breads, cereals, and starches.

### Psychological Qualities

#### Kessler Screening Scale for Psychological Distress

The Kessler Screening Scale for Psychological Distress (K6) [16] consists of 6 questions about an individual’s feelings over the past 2 weeks, including nervousness and hopelessness. Responses range from 1 (“none of the time”) to 5 (“all of the time”); summed scores from 19 to 30 are indicative of high distress [17]. Although the K6 is sensitive to all forms of psychological distress, it does not distinguish between depression and anxiety [16].

#### Perceived Stress Scale

The Cohen Perceived Stress Scale (PSS) measures the perception that demands exceed personal resources to cope. The 10-item version (PSS-10) has high internal consistency and test-retest reliability as well as good construct and predictive validity [18]. The PSS-10 was collected at weeks 0, 26, and 52. The 4-item PSS-4 was collected at all other time points [18].

#### General Self-Efficacy Scale

This validated tool assesses self-efficacy, the belief that one can function under adversity [19]. The 10-item General Self-Efficacy Scale-10 was used at weeks 0, 26, and 52. The shorter General Self-Efficacy Scale-6 collected data at all other time points.

#### Health Anxiety Inventory

This 14-item measure of concerns about health was administered at weeks 0, 26, and 52 [20]. Individuals with hypochondriasis score higher than physically ill patients, individuals with anxiety disorders, and healthy controls [20].

#### Intolerance of Uncertainty Scale

This 12-item survey assesses a tendency toward negativity when faced with uncertain situations. Items are evaluated on a 5-point scale with endpoints of “not at all characteristic of me” and “entirely characteristic of me” [21]. Good convergent and discriminant validity as well as internal consistency have been demonstrated [22,23].

#### Optimism

The Life Orientation Test-Revised is the 10-item optimism survey most commonly used in health research [24]. Each item is scored on a 5-point scale from “strongly disagree” to “strongly agree” [25].

### Inflammatory Bowel Disease Symptom Activity and Health-Related Outcomes

#### Inflammatory Bowel Disease Symptom Inventory

The newly-developed IBD Symptom Inventory (IBDSI) is a 35-item self-report measure assessing 5 symptom clusters: bowel symptoms, abdominal discomfort, fatigue, bowel complications, and systemic complications [26]. It embeds the Harvey-Bradshaw Index for CD [10] and the Powell-Tuck Index for UC [27], so these scores can be calculated from the IBDSI. The complete IBDSI was collected at weeks 0, 26, and 52. A short form of the IBDSI, with 25 items, was collected at all other measurement points.

#### Manitoba Inflammatory Bowel Disease Index

The Manitoba Inflammatory Bowel Disease Index, developed and validated as part of the Manitoba IBD Cohort Study [28], uses a single item to describe symptom persistence. At weeks 0, 26, and 52, participants answered, “In the past 6 months my disease has been (1) constantly active, giving me symptoms every day; (2) often active, giving me symptoms most days; (3) sometimes active, giving me symptoms on some days (for instance 1-2 days per week); (4) occasionally active, giving me symptoms 1-2 days per month; (5) rarely active, giving me symptoms on a few days in the past 6 months; or (6) I was well in the past 6 months, what I consider a remission or absence of symptoms.” Active disease can be defined as experiencing symptoms constantly to occasionally (responses 1-4).

#### Inflammatory Bowel Disease Symptom Change Indicator

This 7-point indicator of symptom change was developed by our group based on the Clinical Global Impression measure [29]. The IBD SCI asks, “Compared to 2 weeks ago, my IBD symptoms are: (1) much improved, (2) moderately improved, (3) minimally improved, (4) no change, (5) minimally worse, (6) moderately worse, or (7) much worse.” A response of “moderately worse” or “much worse” defined symptom exacerbation in this study.

#### Flare Certainty Indicator

At every measurement occasion except week 0, individuals were asked: “Do you consider yourself to be in an IBD disease flare?” Response options included: “I am not in an IBD flare, I am possibly in an IBD flare, I am probably in an IBD flare,” and “I am definitely in an IBD flare.” This scale was created by our research group for this study.

#### Short Inflammatory Bowel Disease Questionnaire

Disease-specific quality of life was measured using the Short Inflammatory Bowel Disease Questionnaire (SIBDQ) [30]. Use of the Inflammatory Bowel Disease Questionnaire (IBDQ), authored by Jan Irvine, MD (1994), was made under license from McMaster University, Hamilton, Canada [31]. The SIBDQ [30,32], based on the original 32-item Inflammatory Bowel Disease Questionnaire, is a 10-item scale measuring bowel, systemic, social, and emotional aspects of living with IBD. It is responsive to changes in disease activity [32].

#### Disability

Questions were adapted from the Canadian Community Health Survey [33] and the Sheehan Disability Scale [34]. Examples are, “How many days in the last 2 weeks did your IBD symptoms cause you to miss work?” and “How many days in the last 2 weeks did you feel so impaired by your IBD symptoms that, even though you went to work, your productivity was reduced?”

#### Health Care Utilization and Clinical Investigations

Participants were asked to report any hospital admissions, emergency department visits, or physician visits (clinic or office) for their IBD in the prior 2 weeks. Every 3 months they reported any clinical investigations, including colonoscopy, capsule endoscopy, flexible sigmoidoscopy, abdominal magnetic resonance imaging or computerized tomography scan, and barium x-ray.

#### Administrative Health Data

At enrollment, consent was given to link the data from this study with the individuals’ administrative health data. Manitoba Health is the single provincial health insurance provider. All physician visits and hospitalizations since 1984 and all prescription medication dispensed since 1995 can be tracked. Administrative data will confirm the dose and quantity of dispensed IBD medications, including 5-aminosalicylates, corticosteroids, thiopurines, methotrexate, and antitumor necrosis factor agents. The Manitoba administrative prescription drug database contains records of all dispensations for the entire population and captures the date of dispensation, information about the prescriber, drug name, and the dose or quantity dispensed [35]. Hospitalizations and outpatient physician and gastroenterologist visits will be confirmed using hospital discharge abstracts and physician billing claims. As well, we will collect utilization and medication information for 1 year prior to study entry.

#### Intestinal Inflammation

At weeks 0, 26, and 52 and at the report of symptom exacerbation, stool specimens were collected to measure FCAL, a marker of intestinal inflammation. Recently, it has been shown that the level of FCAL in a stool sample correlates with the presence and severity of gut inflammation [36-38]. Stool collection kits were delivered and returned by courier to the IBD Clinical and Research Centre, Manitoba, Canada. FCAL levels remain stable for 7 days at room temperature. Once the kit was received at the study center, it was kept at −80℃ until analyzed with a calprotectin enzyme-linked immunosorbent assay (ALPCO, Salem, NH, USA). We obtained 2 measurements from each sample, and the average FCAL level was used for analysis. The upper limit of measurement for FCAL is 1,888 μg/g stool. Participants were considered to have active intestinal inflammation if FCAL exceeded 250 μg/g stool [39,40].

#### Inflammatory Bowel Disease Medications

Participants were asked to select from a list the IBD medications they currently used. They were asked about amounts (eg, mg or mL), the number of pills taken at a time, and timing (eg, 2 times per day). For each medication, participants were asked to report 1 of the following 3 options: (1) number of times the medication was taken in the last 2 weeks, if medication was taken on an as needed basis; (2) percent of scheduled medication taken in the last 2 weeks (eg, 80%); or (3) not scheduled to take medication in the last 2 weeks. Participants were asked to report if they had stopped their IBD medication(s) in the last 2 weeks, or they could report not using any IBD medications.

#### Non-IBD Medications

Every 2 weeks, participants reported if they had started, stopped, or changed the dosage of any medication taken for any reason other than IBD. A full list of all prescribed and nonprescribed medications they used was collected at weeks 0, 14, 26, 40, and 52 as well as when a medication start, stop, or change was reported. Additional multiple-choice questions collected information on amounts of acetaminophen, narcotics, aspirin, and other nonsteroidal anti-inflammatory drugs taken over the 2-week period (0 pills, 1-5 pills; 6-13 pills; 14-28 pills; >28 pills).

#### Medication Adherence Report Scale

The tendency to take medication as prescribed was assessed with the 5-item Medication Adherence Report Scale [41,42], which asks about the propensity to avoid, forget, or stop taking medication and the tendency to alter the dose. Items were scored on a 5-point scale, with response options of “always, often, sometimes, rarely, or never,” and summed, with a total score of 25 indicating complete adherence. The scale can be analyzed either continuously or categorically, with a score of ≥20 categorized as high medication adherence [43].

#### Triggers of Symptom Exacerbation

At baseline and study completion, participants were asked open-ended questions, followed by specific questions, about their experiences with triggers of symptom exacerbations. Specified triggers included stomach or bowel infection, other infections, overwork, sleep problems, conflict, stress, medication changes, more alcohol consumption than usual, overeating, eating certain foods, and not eating certain foods. Participants were asked, “How much has [specified trigger] been related to your IBD symptoms going from inactive to active in the past?” Response options were, “not at all, a little, moderately, quite a lot, and a great deal.” A supplemental survey was sent to participants when they reported at least moderate worsening of symptoms. It asked first, “Do you believe that there was something that triggered your increase in IBD symptoms?” If yes, they were asked to specify. Cases and controls were asked about changes in diet and whether they had been overworked, stressed, down, or anxious.

### Statistical Analysis

We describe the demographic and clinical characteristics of the sample at enrollment using frequencies, percentages, means, and SDs (Table 1). The frequency of individuals who reported at least moderate symptom worsening with the IBD SCI is stated.

Patient attributes, such as age, sex, baseline disease activity, and baseline level of inflammation as measured by FCAL level, will be tested for their association with adherence to the protocol. This analysis will help to inform future studies, to maximize subject participation in IBD studies that use longitudinal methods.

We will track the relationship between symptom exacerbation without active inflammation as measured by FCAL, symptom exacerbation with active inflammation, active inflammation without symptom exacerbation, and how these different combinations of symptoms and inflammation change over time. We will compare self-reported symptom exacerbation on the IBD SCI with symptom scores on the IBDSI as well as with the SIBDQ and individual items from the SIBDQ, such as fatigue and quality of sleep.

Multilevel Poisson regression will model the total number of symptom exacerbations, with and without inflammation. Model covariates will include age, sex, disease type, disease duration, baseline measures of stress, and disease activity. The model fit will be assessed using likelihood ratio statistics and penalized measures of the likelihood function, such as the Aikake information criterion [44]. Potential collinearity among the explanatory variables will be assessed using descriptive correlational analyses and variance inflation factors, where appropriate.

The trajectory of symptom exacerbations (ie, exacerbation present vs absent) over the 12-month observation period will be modeled using a mixed-effects multiple logistic regression model. The model will include both marginal (ie, average) and subject-specific (ie, random) effects, to account for the clustering of repeated observations within participants. Mixed-effects models are widely recommended for longitudinal analyses because of the opportunity to describe both within-subject and between-subject variations across multiple measurement occasions [45,46]. A random intercept for time will be included in the model, and we will explore improvements in model fit when random slopes for time and other covariates are included. Both time-varying and time-invariant covariates will be considered for model inclusion, and time-lagged covariates (eg, perceived stress in the prior 2-week period, perceived stress for all previous 2-week periods, and change in perceived stress between the baseline and prior 2-week period) will be considered. Other covariates will include K6 scores; adherence to maintenance medications; use of nonsteroidal anti-inflammatory drugs, antibiotics, and other non-IBD medications; smoking status; and most recent FCAL value. The model fit will be evaluated using the methods noted above. The intraclass correlation will be used to describe the proportion of model variation due to subject-specific effects.

We will assess diet stability in persons with IBD by comparing their responses to the FFQ at week 2 with the FFQ at week 50. More importantly, the stability in diet between those persons who reported a symptom exacerbation will be compared to those who did not report an exacerbation. Furthermore, we will determine if the frequency of foodstuff consumption differs between persons who had a symptom exacerbation but normal FCAL compared to persons who had an exacerbation with active inflammation as identified by FCAL.

**Table 1:** Demographic and disease characteristics of participants at baseline (N=155).

Characteristics		Value
Female, n (%)		108 (69.7)
Age, mean (SD)		42.6 (12.6)
European or Canadian ethnicity, n (%)		149 (96.1)
Urban residence, n (%)		132 (85.2)
**Marital status, n (%)**		
	Married or living as married	97 (62.6)
	Separated, divorced, or widowed	22 (14.2)
	Single or never married	36 (23.2)
Education (years), mean (SD)		15.6 (3.5)
Crohn disease, n (%)		102 (65.8)
Age at diagnosis (years), median		28
Age at diagnosis (years), mean (SD)		28.6 (11.5)
**Age (years), n (%)**		
	<17 years	23 (14.8)
	17-39 years	107 (69.0)
	≥40 years	25 (16.1)
Disease duration (years), mean (SD)		14.8 (10.3)
Comorbid chronic condition, n (%)		91 (58.7)
Previous IBD^a^-related surgery, n (%)		55 (35.5)
IBD-related hospitalization in the past year, n (%)		19 (12.3)
Current smoker, n (%)		28 (18.1)
Alcohol consumption >14 drinks per week, n (%)		3 (1.9)
**Active disease at baseline, n (%)**		
	IBDSI^b^ at baseline: >24 in Crohn disease, >17 in ulcerative colitis	74 (47.7)
	Fecal calprotectin at baseline, >250 μg/g	71 (45.8)
Symptom exacerbation during the study, n (%)		74 (47.7)

^a^IBD: inflammatory bowel disease.

^b^IBDSI: Inflammatory Bowel Disease Symptom Index.

Among those persons who reported a symptom exacerbation, we will assess whether a change in diet occurred in the 2 weeks prior. This will be done using a supplemental food survey that asks about specific food items that were consumed “more than usual in the last 2 weeks” (eg, types of milk products, grains, fruits, and vegetables) as well as whether the change was made prior to the flare or as a response to the symptoms. This supplemental survey was administered when a participant reported a symptom exacerbation on the SCI.

With our sample size of 155 and 15 predictor variables in a regression model, with alpha=.05 and power of 80%, we can detect an effect that accounts for at least 12% of the variation in a continuous outcome measure. With a model that includes 5 predictor variables, with alpha=.05 and power of 80%, we can detect an effect that accounts for at least 8% of the variation in a continuous outcome measure.

## Results

Between June 2015 and May 2017, 158 individuals were enrolled. Of this number, 3 were later withdrawn: 1 did not meet medical exclusion criteria (diabetes requiring insulin), and 2 returned their initial stool sample but did not complete a baseline survey and were lost to follow-up. Therefore, the final study population included 155 participants with IBD: 66.5% (103/155) with CD and 33.5% (52/155) with UC. Of these, 89.7% (139/155) completed the study to 52 weeks and completed at least 80% of all surveys.

**Table 2:** Recruitment sources for study participants.

Recruitment source	n (%)
Gastroenterology clinic	119 (76.8)
Manitoba IBD^a^ Cohort Study or previous research program	22 (14.2)
Manitoba IBD Research Registry	9 (5.8)
Poster or Crohn's and Colitis Canada article	5 (3.2)

^a^IBD: inflammatory bowel disease.

**Table 3:** Comparison of the Living with IBD Study and the University of Manitoba IBD Epidemiology Database (UMIBDED).

Measure	Study participants (N=155)	UMIBDED (N=10,636)
Female, n (%)	108 (69.68)	5684 (53.44)
Crohn disease, n (%)	102 (65.81)	5241 (49.28)
Urban residence, n (%)	132 (85.16)	6518 (61.28)
Median age at diagnosis (years)	28	36

Over the course of the 1-year study period, 47.7% (74/155) participants experienced a symptom exacerbation and were matched to a control who had not previously reported a symptom exacerbation. There were 32 participants who had >1 exacerbation (see Table 1 for participant demographics and clinical descriptions).

Participants were recruited from multiple sources (Table 2), most from gastroenterology clinics, reflecting the requirement of symptoms related to IBD within the last 2 years. Recruitment averaged 6.4 persons per month over 24 months. When compared to the provincial population of Manitobans with IBD, according to the University of Manitoba IBD Epidemiology Database, this study enrolled a higher proportion of females, persons with CD, and persons living in an urban center (Table 3).

## Discussion

By surveying persons with IBD every 2 weeks over the course of 1 year and analyzing exacerbation cases compared with matched controls, we aim to gain insight into factors associated with exacerbation and the extent to which symptoms are associated with active inflammation. Ultimately, we hope our findings can improve the care of persons with IBD by better understanding the trajectory of the disease and the factors that affect its course.

Difficulties in the past in investigating the relationship between symptom exacerbations and potential triggers have been primarily methodological. Multiple factors across biological and psychological domains have rarely been assessed concurrently or assessed frequently enough to establish a contributory role. Hence, we anticipate that our study will substantially advance clinical knowledge. Much of the research to date has relied on the retrospective survey of patients with IBD, where they are asked about the presence and the severity of symptoms along with their history of exposure to potential triggers. We have also used this method previously [4,47]. While we reported that, of all factors monitored over a 3-month period, only perceived stress was associated with an exacerbation of symptoms, the retrospective rating of symptoms and potential triggers over 3-month periods may be subject to recall bias. Recall biases may also influence how participants assessed the temporality and directionality of the relationship between triggers and symptoms. Assessments carried out at more frequent intervals allow for a more accurate assessment of the presence of triggers, the severity of symptoms, and the temporal relationship between them.

The relationship between patient symptoms and inflammation is central to disease management as most of the medication options for the treatment of IBD symptom exacerbations involve either the intensification or modification of anti-inflammatory therapies [48]. Conversely, it has been shown that persons with quiescent IBD symptoms who have evidence of ongoing inflammation may be at higher risk of developing a symptom exacerbation over the next 2 years. The measurement of FCAL may play a significant role in noninvasively studying the association between inflammation and symptom exacerbation. Determining the relationship between intestinal inflammation and symptoms could better define pathophysiologic mechanisms. Understanding the symptom-inflammation relationship would also better inform the management of symptom flares, given options of initiation or intensification of anti-inflammatory therapy or, alternatively, use of supportive symptom-directed care. Feedback to patients about the relationship between symptom presentation and measurable inflammation may also increase their confidence in using self-management approaches and knowing when to contact their physician for medical intervention.

The high retention rate of 98.7% (153/155) is a strength of our study. Staggered financial incentives [49], frequent appreciative written communication with participants as well as prompt and repeated electronic and phone call reminders may have contributed to the high retention. The use of electronic methods in this study was a strength in maintaining high participation for the entire 52 weeks as well as for having data entered into a secure, analyzable database directly by the participants as they completed their surveys.

There were some limitations to our study. Women were overrepresented, similarly to other IBD cohort studies [4,47]. FCAL is a sensitive measure of intestinal inflammation in persons with colitis but may be less sensitive in persons with small bowel CD [50].

In conclusion, we have reported the protocol and enrollment for a longitudinal study of persons with IBD reporting on their lived experiences biweekly for 1 year as well as intermittently providing stool samples to study intestinal inflammation. We believe our study will provide a unique assessment of the course of IBD. We hope to define factors that are associated with triggering a symptom exacerbation. This will help both clinicians and patients better understand how to manage IBD.

## Appendix

Multimedia Appendix 1 Timetable of measure administration.

## Abbreviations

CD: Crohn disease
FCAL: fecal calprotectin
FFQ: Food Frequency Questionnaire
IBD: inflammatory bowel disease
IBDSI: Inflammatory Bowel Disease Symptom Index
K6: Kessler Screening Scale for Psychological Distress
PSS: Perceived Stress Scale
SCI: Symptom Change Indicator
SIBDQ: Short Inflammatory Bowel Disease Questionnaire
UC: ulcerative colitis
