# Supplementary material for: Living With Inflammatory Bowel Disease: Protocol for a Longitudinal Study of Factors Associated With Symptom Exacerbations
Source: JMIR Res Protoc. 2018 Nov 12;7(11):e11317. doi: 10.2196/11317 (PMC6256106; doi:10.2196/11317)
Supplement: Multimedia Appendix 1 [file resprot_v7i11e11317_app1.pdf]

Multimedia Appendix 1. Living with Inflammatory Bowel Disease (IBD) timetable of measure administration.

**Measure**	**Week**																										
	**0**	**2**	**4**	**6**	**8**	**10**	**12**	**14**	**16**	**18**	**20**	**22**	**24**	**26**	**28**	**30**	**32**	**34**	**36**	**38**	**40**	**42**	**44**	**46**	**48**	**50**	**52**
**Demographics**	✓																										
**IBD Disease Characteristics**	✓																										
**Chronic Conditions**	✓																										✓
**Previous Non-IBD surgery**	✓																										
**Health:** new illness or injury or worsening of chronic condition		✓	✓	✓	✓	✓	✓	✓	✓	✓	✓	✓	✓	✓	✓	✓	✓	✓	✓	✓	✓	✓	✓	✓	✓	✓	✓
**Menstruation** (if reported at baseline)	✓	✓	✓	✓	✓	✓	✓	✓	✓	✓	✓	✓	✓	✓	✓	✓	✓	✓	✓	✓	✓	✓	✓	✓	✓	✓	✓
**Pregnancy** (if menstruation was reported at baseline)																											✓
**Cigarettes and Alcohol**	✓																										
**Diet:** Harvard Food Frequency Questionnaire		✓																								✓	

	0	2	4	6	8	10	12	14	16	18	20	22	24	26	28	30	32	34	36	38	40	42	44	46	48	50	52
**Psychological Distress** (K6)	✓	✓	✓	✓	✓	✓	✓	✓	✓	✓	✓	✓	✓	✓	✓	✓	✓	✓	✓	✓	✓	✓	✓	✓	✓	✓	✓
**Psychological Stress** (PSS-10)	✓													✓													✓
**Psychological Stress** (PSS-4)		✓	✓	✓	✓	✓	✓	✓	✓	✓	✓	✓	✓		✓	✓	✓	✓	✓	✓	✓	✓	✓	✓	✓	✓	
**General Self-Efficacy** (GSES-10)	✓													✓													✓
**General Self-Efficacy** (GSES-6)		✓	✓	✓	✓	✓	✓	✓	✓	✓	✓	✓	✓		✓	✓	✓	✓	✓	✓	✓	✓	✓	✓	✓	✓	
**Health Anxiety** (HAI)	✓													✓													✓
**Intolerance of Uncertainty** (IUS-12)	✓													✓													✓
**Optimism** (LOT-R)	✓													✓													✓
**Inflammation:** fecal calprotectin	✓													✓													✓
**Triggers of Symptom Exacerbation**	✓																										✓

	0	2	4	6	8	10	12	14	16	18	20	22	24	26	28	30	32	34	36	38	40	42	44	46	48	50	52
**IBD Symptom Inventory Long Form** (IBDSI-LF)	✓													✓													✓
**IBD Symptom Inventory Short Form** (IBDSI-SF)		✓	✓	✓	✓	✓	✓	✓	✓	✓	✓	✓	✓		✓	✓	✓	✓	✓	✓	✓	✓	✓	✓	✓	✓	
**Manitoba IBD Index** (MIBDI)	✓													✓													✓
**IBD Symptom Change Indicator** (SCI)		✓	✓	✓	✓	✓	✓	✓	✓	✓	✓	✓	✓	✓	✓	✓	✓	✓	✓	✓	✓	✓	✓	✓	✓	✓	✓
**Flare Certainty Indicator**		✓	✓	✓	✓	✓	✓	✓	✓	✓	✓	✓	✓	✓	✓	✓	✓	✓	✓	✓	✓	✓	✓	✓	✓	✓	✓
**Active vs Inactive Disease**		✓	✓	✓	✓	✓	✓	✓	✓	✓	✓	✓	✓	✓	✓	✓	✓	✓	✓	✓	✓	✓	✓	✓	✓	✓	✓
**Short IBD Questionnaire** (SIBDQ)	✓	✓	✓	✓	✓	✓	✓	✓	✓	✓	✓	✓	✓	✓	✓	✓	✓	✓	✓	✓	✓	✓	✓	✓	✓	✓	✓
**Disability**		✓	✓	✓	✓	✓	✓	✓	✓	✓	✓	✓	✓	✓	✓	✓	✓	✓	✓	✓	✓	✓	✓	✓	✓	✓	✓
**Health Care Utilization**		✓	✓	✓	✓	✓	✓	✓	✓	✓	✓	✓	✓	✓	✓	✓	✓	✓	✓	✓	✓	✓	✓	✓	✓	✓	✓
**IBD Investigations**	✓							✓						✓							✓						✓

	0	2	4	6	8	10	12	14	16	18	20	22	24	26	28	30	32	34	36	38	40	42	44	46	48	50	52
**IBD Medications**	✓	✓	✓	✓	✓	✓	✓	✓	✓	✓	✓	✓	✓	✓	✓	✓	✓	✓	✓	✓	✓	✓	✓	✓	✓	✓	✓
**Non-IBD Medications:** full list	✓							✓						✓							✓						✓
**Non-IBD Medications:** changes		✓	✓	✓	✓	✓	✓	✓	✓	✓	✓	✓	✓	✓	✓	✓	✓	✓	✓	✓	✓	✓	✓	✓	✓	✓	✓
**Pain Medications**		✓	✓	✓	✓	✓	✓	✓	✓	✓	✓	✓	✓	✓	✓	✓	✓	✓	✓	✓	✓	✓	✓	✓	✓	✓	✓
**Medication Adherence** (MARS-5)	✓																										✓

Witges K, Targownik LE, Haviva C, Walker JR, Graff LA, Sexton KA, Lix L, Sargent M, Vagianos K, Bernstein CN
Living With Inflammatory Bowel Disease: Protocol for a Longitudinal Study of Factors Associated With Symptom Exacerbations
JMIR Res Protoc 2018;7(10):e11317
http://www.researchprotocols.org/2018/10/e11317/
